# Organic Analysis of Peridotite Rocks from the Ashadze and Logatchev Hydrothermal Sites

**DOI:** 10.3390/ijms10072986

**Published:** 2009-07-03

**Authors:** Marie-Paule Bassez, Yoshinori Takano, Naohiko Ohkouchi

**Affiliations:** 1 Département Chimie, IUT, Université de Strasbourg, 72 route du Rhin, 67400 Illkirch, France; 2 Institute of Biogeosciences, Japan Agency for Marine-Earth Science and Technology (JAMSTEC), 2–15 Natsushima, Yokosuka 237-0061, Japan; E-Mails: takano@jamstec.go.jp (Y.T.); nohkouchi@jamstec.go.jp (N.O.)

**Keywords:** origin of life, prebiotic synthesis, organic geochemistry, physical-chemistry, exobiology

## Abstract

This article presents an experimental analysis of the organic content of two serpentinized peridotite rocks of the terrestrial upper mantle. The samples have been dredged on the floor of the Ashadze and Logatchev hydrothermal sites on the Mid-Atlantic Ridge. In this preliminary analysis, amino acids and long chain *n*-alkanes are identified. They are most probably of biological/microbial origin. Some peaks remain unidentified.

## 1. Introduction

The origin of terrestrial life is not yet understood. An accepted hypothesis is that a transition occurred between a molecular prebiotic evolution and a biological evolution and that prebiotic organic matter could have been delivered to Earth within carbonaceous chondrite meteorites, such as the CM2 Murchison meteorite.

At the bottom of the terrestrial oceans, where tectonic forces separate the lithospheric plates along mid-ocean ridges, the ultramafic rocks of the upper-mantle, the peridotites, are exposed to circulating seawater [1]. They encounter various physico-chemical conditions and the hydrolysis of their silicate constituents, the olivine and pyroxenes minerals, into serpentine, occur at different degrees of serpentinization depending on the characteristics of the medium: temperature, pressure, oxygen fugacity, nature and composition of the fluid phase, fluid flux, pH, rock composition, water:rock ratio [2]. The Mid-Atlantic-Ridge, MAR, is covered with several hydrothermal sites and presents black smoker activity. The active Logatchev site, 14° 45′N–43′N, at a water depth of 2,970 m and the active Ashadze site (12° 58′N, 4,080 m) are located on an ultramafic geological environment of serpentinized peridotite rocks, while the Krasnov site (16° 38′N), discovered with the Ashadze site during the 2007 French-Russian Serpentine cruise [3] is inactive and located on a basaltic environment. Ultramafic environments seem enriched in Cu and Zn content compared to the basaltic ones [3].

The Logatchev hydrothermal *vent fluids* originate from the interaction between the underlying peridotite rocks and seawater. They have been previously analyzed [4]. The H_2_ concentration is 12 mmol/wkg (data from 1996) and 19 mmol/wkg (data from 2005) and the analyses made in 1996, 2004 and 2005 show a stable composition of the fluids. The analyses of the Ashadze vent fluids [5] also show a great amount of H_2_. Both these vent fluids, as those of the Rainbow site (36° 14′N on the MAR, 2,300 m) also contain significant amounts of CO_2_, CH_4_, N_2_, CO. Their pH is acidic ~3–4, the temperature of their fluids is ~310-370 °C and the detected saturated hydrocarbons, carboxylic acids and methyl esters in the fluids have been proposed of either abiogenic origin or not [5,6].

An accepted *hypothesis* to explain the occurence of the *carbon-based organic compounds* in the fluids is the synthesis of these molecules in the context of catalytic Fischer-Tropsch Type (FTT) reactions involving hydrothermal CO_2_. The dihydrogen, formed during the hydrolysis of the peridotite terrestrial rocks, which contain ferrous iron-rich minerals, olivine and pyroxenes, could react with hydrothermal CO_2_, to form methane and saturated hydrocarbons. Hydrocarbons have been synthesized during *experimental serpentinization* of olivine at 300 °C and 500 bar [7] and methane, ethane and propane were synthesized at 390 °C and 400 bar in an experiment catalyzed with Cr_2_O_3_ in combination with FeO [8]. A more recent experiment, at 200 °C and 500 bar, simulating subseafloor serpentinization produced significant amounts of dissolved H_2_ when artificial seawater reacted with a peridotite rock composed of 62% olivine, 26% orthopyroxene and 10% clinopyroxene. Even during the early stages of the reaction, ~25 mmol/kg of water are produced after 2,000 h of experiment and 77 mmol/wkg after 8,000 h [9]. Experiments conducted at 250 °C and 325 bar on an aqueous solution of formic acid (HCOOH) in the presence of Fe produced a series of *n*-alkanes with typical FTT distribution. Volatile hydrocarbons (C_1_–C_6_), magnetite (Fe_3_O_4_) and siderite FeCO_3_ were also detected [10]. FTT mechanism can be invoked since hydrothermal Fe reacts with water to form magnetite and H_2_ and formic acid decomposes into CO_2_ and H_2_.

The exact factors that control the hydrolysis of peridotite remain unknown. Calculations considering the thermodynamics of fluid mixing between hydrothermal fluids containing dissolved CO_2_ and H_2_ at 350 °C, and seawater containing bicarbonate at 2 °C, led to the organic synthesis of carboxylic acids, alcohols, ketones [11]. These calculations depend on the fugacity of O_2_. They show that the oxidation state of ultramafic rocks, driven by the equilibrium of the FMQ, fayalite-magnetite-quartz mineral assemblage, lead to a lower oxygen fugacity and a greater potential for organic synthesis than for the PPM, pyrrhotite-pyrite-magnetite assemblage. Numerical models, considering a rock composed of 80 wt% olivine, 15 wt% orthopyroxene and 5 wt% clinopyroxene predict that, at 35 MPa, a peak production of H_2_ (a few hundred mmol/kg) occurs approximately at temperatures of 200–315 °C. These models also predict a decrease in pH from ~11 to ~6, when the temperature increases from 50 °C to 400 °C, with pH values of ~9 around 150 °C and ~8 around 200 °C [12].

Analyses of hydrothermally altered peridotites drilled between 14°N and 16°N on the Mid-Atlantic Ridge (MAR) between 1,800 and 4,000 m depth have been reported [13]. They suggest that extensive serpentinization processes occur at all sites and that the transformation of the mineral olivine into serpentine, magnetite and brucite with release of H_2_ is favored at temperatures below 250 °C, while pyroxene is replaced by talc and tremolite above 350–400 °C [2,13], where olivine is stable. The latitude of these drillings corresponds to the area of the hydrothermal sites Logatchev, Ashadze and Krasnov.

Several experiments have demonstrated the production of hydrothermal organic matter including nitrogen atoms at various temperatures, 100–400 °C, and pressures and with various starting compounds [14–20 and Ref. therein]. A gas mixture of methane and dinitrogen above simulated seawater under ~8 MPa at room temperature was heated to 325 °C. Amino acids were extracted after acid hydrolysis of the products [15]. In experiments conducted at 150 °C and 1 MPa with HCN, CH_2_O, NH_3_ in the presence of the PPM redox buffer, amino acids were also detected [16]. Their yields were higher than in previous gaseous spark discharge experiments [14]. Di- and triglycine were synthesized in a flow reactor under 24.0 MPa at 200 °C–250 °C with consecutive quenching at 0 °C. The presence of copper ions seemed to help synthesize tetraglycine [17]. Using a supercritical water flow reactor with temperature control inside the fluids, it is suggested that condensates of glycine, which yielded amino acids after hydrolysis, formed even in supercritical water at 400 °C, under 25 MPa pressure [18]. When an aqueous mixture of ten amino acids was heated at 200–400 °C, the acid hydrolysis of the products led to a higher content in glutamic acid and α-amino acids, such as α-aminobutyric acid, 5-aminovaleric acid and 6-aminohexanoic acid than in α-amino acids even over supercritical conditions of water suggesting that α-amino acids could be chemical markers of abiotic hydrothermal systems [18]. Reviews report the various conditions of amino acid syntheses [19–21 and Ref. therein].

Recent calculations using measured data of the Rainbow hydrothermal site, show that an abiotic synthesis of the five nucleobases and of the two sugars from formaldehyde and hydrogen cyanide is thermodynamically favored between 0 °C and 150–250 °C [22 and Ref. therein].

Some similarities with the Murchison meteorite can be noticed. The Murchison mineral structure is dominated with a phyllosilicate (serpentine) matrix which contains minerals such as olivine, pyroxenes, calcium carbonates, iron oxides (magnetite), iron-nickel sulfides and sulfates [23–25]. It has been altered by water, by heat, by pressure shock waves, by short-lived radionuclides [26,27]. The transformation of olivine and pyroxene chondrules seems to grow with the extent of mineral hydrolysis and the formation of water-soluble organic compounds is described at temperatures below ~125 °C [28,29]. Aside from any terrestrial contamination, all the classes of organic molecules considered of biological relevance are identified [30–32 and Ref. therein] and also non-terrestrial amino acids and enantiomeric excesses [33–35].

Several hypotheses are proposed for the production of meteoritic organic matter, either solar-nebula processes or secondary processes which occurred after the accretion, on asteroidal parent bodies [35–39, 23, 40–46 and Ref. therein]. Among these are FTT reactions; ion-molecule and radical-radical reactions; γ-, proton- and UV-irradiation; Strecker’s type reactions involving aqueous processing of simple molecules such as H_2_O, HCN, H_2_CO and NH_3_; internal heating of the parent body produced by the radioactive decay of short-lived nuclides. Although quite significant amounts of glycine are detected in the Murchison meteorite, no ascertained interstellar glycine has yet been identified since its first observational report in 1979 [47] suggesting that molecules formed in the interstellar medium, ISM, underwent further processing. It has been suggested that the primary products from proton irradiation of a mixture of CO, N_2_/NH_3_, H_2_O are amino acid precursors, molecules that provide amino acids after acid hydrolysis [36,38]. Amino acids recovered after acid hydrolysis of products obtained in vacuum UV-photolysis of H_2_O, CO, CO_2_, CH_3_OH, CH_4_, NH_3_, simulating the ISM, do not match the Murchison meteorite distribution, suggesting that the organic molecules found in the meteorite parent bodies experienced contact with water [46]. Indeed, it has been demonstrated that bound amino acids in aqueous solution exposed to γ- and UV- rays are much more photostable than the corresponding free amino acids [39].

Thus, it seems consequently plausible to imagine that the H_2_, released during the serpentinization processes of the peridotite terrestrial rocks, could react with the CO_2_ embedded inside the rock, to form methane and saturated hydrocarbons, in the context of catalytic reactions involving hydrothermal CO_2_. The simple molecules H_2_O, H_2_, CO_2_, CH_4_, would be present as a consequence of mineral reactions of the terrestrial peridotites with seawater and, with the N_2_ of the environment and with an activation source such as gamma rays, they could form the simple organic molecules of biological relevance [48–50,22,51 and Ref. therein].

These reactions could occur at temperatures ~150–200 °C, where olivine transforms into serpentine, magnetite and brucite with the release of H_2_. At these temperatures, combined with the pressures encountered at the hydrothermal sites, many compounds are in their supercritical state and peculiar chemistry can occur. In this IJMS issue on the Origin of Life, syntheses of amino acids in a mixture of supercritical CO_2_-liquid water (10:1) starting with hydroxylamine hydrochloride and pyruvic or glyoxylic acid are reported [52]. A hypothesis for the origin of the living systems could consequently be found at the bottom of the oceans, in ultramafic hosted hydrothermal systems, where tectonic plates separate to leave the upper mantle rock reacts with seawater to form hydrothermally altered peridotites and lead to the necessary molecules for life to emerge.

In this hypothesis, serpentinized peridotite rocks located on hydrothermal sites could contain organic molecules. Here we report organic analyses made on two peridotite rocks of Ashadze (12° 58′N, 4,080 m) and Logatchev (14° 43′N, 2,970 m) hydrothermal sites in the Mid-Atlantic Ridge. The samples have been dredged on the seafloor in march 2007, during the French-Russian Ifremer Serpentine cruise [53]. These organic analyses provide the first observations of organic compounds in the serpentinized peridotite rocks of Ashadze and Logatchev hydrothermal sites. They are reported here for the first time.

## 2. Experimental Methods

The analyses have been carried out in the Institute of Biogeosciences of the Japan Agency for Marine-Earth Science and Technology, in Yokosuka. The rock sample was pre-washed by ultra-pure methanol to eliminate possible exogenous compounds from the external surfaces. An aliquot of dried and grounded sample powder (*ca* 0.5 g) was dispensed into 16 x 100 mm reaction vials with PTFE-lined caps and acid hydrolyzed with 6 M HCl at 110 °C for 12 h. Non-polar fraction was extracted by liquid/liquid separation in HCl solution and 2.0 mL of a hexane/dichloromethane (6:5, v/v) mixture in two portions. The hexane/dichloromethane fraction was recovered and dried under a gentle nitrogen flow, and then 200 μL of dichloromethane was added to the final non-polar fraction.

Another procedure was used for the polar fraction, especially for amino acids. After drying the hydrolysis residue under N_2_ flow, the samples were adjusted to pH 1 with 0.1 M HCl, and the amino acid fraction was isolated with cation-exchange column chromatography. The purification of amino acid fractions via application to an AG-50W-X8 (200–400 mesh; Bio-Rad Laboratories) cation exchange resin column was performed by the procedure described earlier [46]. Briefly, a slurry of resin in deionized water was poured into a disposable glass pipette column plugged with quartz wool. Before the injection of the sample to the column, the resin was cleaned by passing three bed volumes (resin/carrier, 1:3, v/v) of 1 M HCl, H_2_O, 1 M NaOH, and H_2_O through the column in succession (i.e., 2 mL of AG50 resin requires 6 mL of 1 M HCl for the first prewash). Immediately before the injection of the sample, the resin was reactivated to the H^+^ form with three bed volumes of 1 M HCl and then rinsed with three bed volumes of H_2_O. The sample solution was loaded and then eluted with three bed volumes of H_2_O to retain only the amino acid fraction. Finally, the amino acid fraction was eluted with three bed volumes of 10% NH_3_ aqueous solution, and then dried by nitrogen flow for the next derivatization procedure.

**Figure 1.**
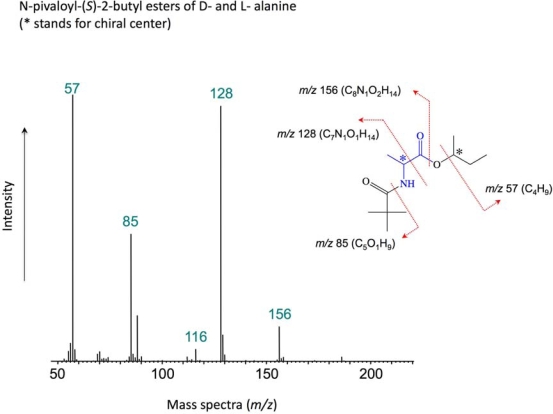
Mass fragment pattern of the *N*-pivaloyl-(S)-2-butyl esters of the d- and l-alanine diastereoisomers.

The esterification reaction was performed with 500 μL of a thionyl chloride/(*S*)-(+)-2-butanol mixture (1:4, v/v) at 110 °C for 2 h. After the solution had been cooled to ambient temperature, it was evaporated to dryness under a gentle nitrogen flow at ~80 °C. The acylation reaction was then performed with 500 μL of a pivaloyl chloride/dichloromethane mixture (1:1, v/v) at 110 °C for 2 h. After cooling, the solution was again evaporated to dryness with a gentle nitrogen flow at ~80 °C. The *N*-pivaloyl-(*S*)-2-butyl esters (NP/*S*2Bu) of the amino acid diastereomers [46] were extracted by liquid/liquid separation in 0.5 mL of distilled water and 1.0 mL of a hexane/dichloromethane (6:5, v/v) mixture for two times. The hexane/dichloromethane mixture fraction containing the NP/*S*2Bu esters was recovered and dried under a gentle nitrogen flow. Then, 200 μL of dichloromethane was added to the final fraction. The NP/*S*2Bu esters of the amino acid diastereomers (Figure 1) were identified by a gas chromatograph/mass spectrometry (GC/MS; Agilent Technologies 6890N/5973MSD). The capillary column used for GC was an HP-5 (30 m × 0.32 mm i.d., 0.52 μm film thickness; Agilent Technologies). The GC oven temperature was programmed as follows: initial temperature 40 °C for 4 min, ramped up at 10 °C min^–1^ to 90 °C, and ramped up at 5 °C min^–1^ to 220 °C, where it was maintained for 10 min. The MS was scanned over *m/z* of 50–550 with the electron-impact mode set at 70 eV. Optically active (*S*)-(+)-2-butanol (purity 99%; boiling point 99–100 °C) was obtained from Sigma-Aldrich Co. All glassware was heated at 450 °C for 4 h before use to eliminate any possible contaminants.

## 3. Results and Discussion

**Figure 2.**
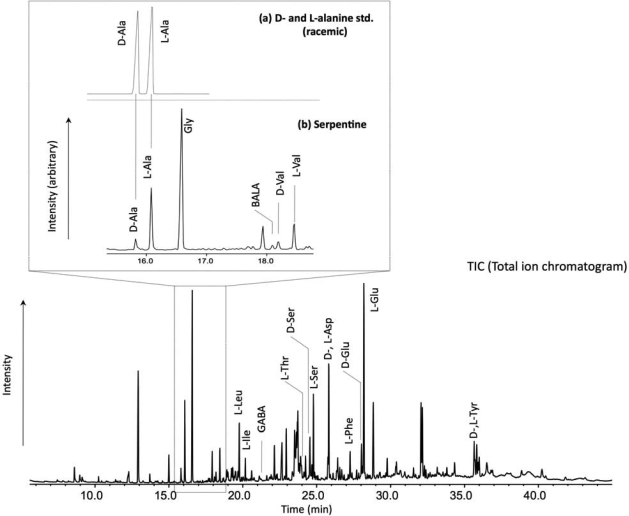
Representative chromatogram of chiral separation for d- and l-amino acids in polar fraction extracted from the Ashadze serpentinized peridotite rock sample by GC/MS analysis. Abbreviations: d-Ala, d-Alanine; l-Ala, l-Alanine; Gly, Glycine; BALA, beta-Alanine; d-Val, d-Valine; l-Val, l-Valine; l-Leu, l-Leucine; l-Ile, l-Isoleucine; GABA, gamma-aminobutyric acid; l-Thr, l-Threonine; d-Thr, d-Threonine; d-Ser, d-Serine; l-Ser, l-Serine; d-, l-Asp, d-, l-Aspartic acid; l-Phe, l-Phenylalanine; d-Glu, d-Glutamic acid; l-Glu, l-Glutamic acid; d-,l-Tyr, d-,l-Tyrosine.

As seen in Figure 2, we identify a wide variety of amino acids including protein and non-protein amino acids. Among these, glycine and glutamic acid are more predominant than the others. Although non-proteinous amino acids such as sarcosine, beta-alanine (BALA) and gamma-aminobutyric acid (GABA) have been found as products in laboratory experiments simulating hydrothermal systems [15], in our experiment sarcosine is under detection limit and BALA and GABA are present as minor constituents. The peak at 17.9 min could not be identified. Figure 1 illustrates the mass spectrum of the *N*-pivaloyl-(*S*)-2-butyl esters obtained for the identification of the d- and l-alanine of the gas chromatogram (Figure 2). It corresponds to the retention times of the alanine peaks in the chromatogram.

**Figure 3.**
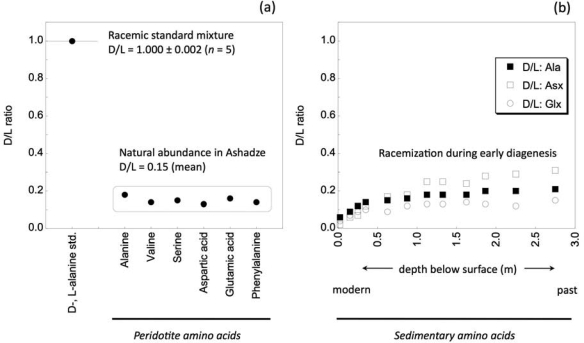
d/l amino acid ratios in the analysed Ashadze peridotite rock and in sedimentary rocks.: Ala (alanine), Asx (asparagine and aspartate) and Glx (glutamine and glutamate).

For amino acids formed abiotically [15], the d/l ratio of amino acids converges to around 1. On the other hand, large enantiomeric excess of l-form amino acids may indicate that the amino acids are derived from sub-seafloor biogenic processes [54] or abiogenic racemization reaction during the pathway of stereochemical conversion via alpha-hydrogen elimination. The racemization of amino acid standards during 22 hours hydrolysis treatment ranged 0.5–1.3% for d-alanine generated from l-alanine [55]. Here, as seen in Figure 3a for the Ashadze peridotite rock, the molar fraction (%d- and %l-) of d-alanine: l-alanine in the serpentine sample is 15:85, hence D/L ratio is 0.18 and other amino acids are also l-form predominant. On Figure 3b the D/L ratios of the sedimentary amino acids, Ala, Asx (asparagine and aspartate) and Glx (glutamine and glutamate) shows the racemization process during early diagenesis as a function of depth over 10,000 years [56]. The similarities in the values on D/L ratios provide a plausible conclusion of a biological origin for the amino acids identified in the Ashadze peridotite sample and also for the Logatchev sample. Although the prokaryotic community in hydrothermal sediments of the Alvin zone location, ~3,500 m, near the TAG mound, ~26°N on the MAR, seems present with a low total cell count [57], we conclude in a biological origin for the identified amino acid peaks.

**Figure 4.**
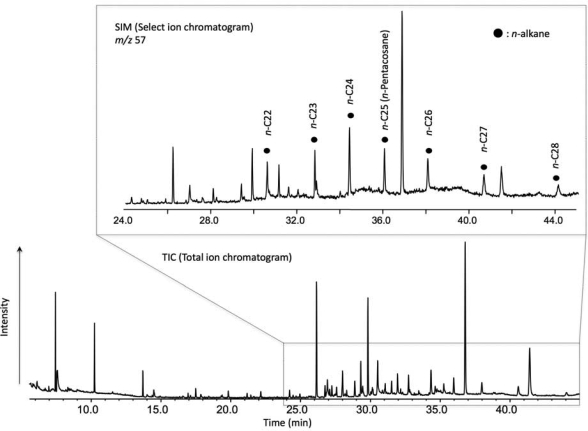
Representative chromatogram of hydrocarbons including *n*-alkanes in non-polar fraction of the Logatchev rock sample. Select ion monitoring (SIM) was also performed to identify *n-*alkane chain analogs.

We also detect a long-chain *n*-alkane compound (< *n*-C_28_H_58_) in the non-polar fraction (Figure 4) under GC conditions up to 220 °C. Although we do not identify lipid compounds in this non-polar fraction, long-chain *n*-alkanes may have two origins. One can be fossilized past biota and/or present microbes which migrated within hydrothermal fluids and the other can be hydrothermally synthesized and/or altered organic molecules.

The GC/MS of the *n*-alkanes shows a decrease in intensity with increasing carbon number, which seems to be a characteristic of abiotic synthesis [10]. Recently, an abiogenic hydrocarbon production by FTT at Lost City hydrothermal field has been proposed wherever warm ultramafic rocks are in contact with water [58]. However, as discussed for the Suiyo Seamount, Izu-Bonin Arc, Pacific Ocean [54] and for the Lost City, Mid-Atlantic Ridge [59] hydrothermal systems, it is difficult to differentiate biotic/abiotic sources. An experimental analysis of the isotopic fractionation of the stable carbon-13 and carbon-12 elements in the organic compounds detected in the Ashadze and Logatchev samples would, as it is widely thought, indicate if these organic compounds derive from microbial decomposition or from an abiotic synthesis. However, it has been demonstrated in laboratory experiments conducted at 250 °C and 350 bar, that organic products, synthesized abiotically in FTT reactions, are depleted in ^13^C to a degree typically ascribed to biological processes [10]. These experiments indicate that the analysis of the carbon isotopic fractionation is an ineffective diagnostic to distinguish between abiotic and biotic origin of organic compounds. Consequently, we will not proceed to the carbon isotopic analysis of the rocks and we do not conclude yet in a biotic or abiotic origin for the identified *n*-alkanes.

## 4. Conclusions

This preliminary analysis of the organic composition of two peridotite rock samples dredged on the ocean floor of the Logatchev and Ashadze hydrothermal sites on the Mid-Atlantic Ridge allows the identification of amino acids and long-chain *n*-alkanes. Many peaks of the amino acid gas chromatograms remain unidentified. Further analyses need to be made with non terrestrial amino acids as references. Signals of abiotically formed organic compounds may be present with negligible intensity compared to the intensities of the identified biotical signals. Consequently, we conclude in a biotic origin for the identified amino acids but we do not exclude an abiotic origin for some amino acids which correspond to the not yet identified peaks. Especially because it is difficult to conclude anything about a biotic/abiotic origin for the *n*-alkanes, since carbon isotopic fractionation is inefficient in distinguishing these sources. It would be more appropriate to analyze samples which are drilled far beneath the ocean floor and which would be less exposed to biological contamination. That could be one goal of a next IODP (Integrated Ocean Drilling Program) cruise.
